# P-560. Evaluating the Use of Long-Acting Lenacapravir among Treatment-Experienced Adults with HIV in a Large Community-Based Clinic Network

**DOI:** 10.1093/ofid/ofae631.759

**Published:** 2025-01-29

**Authors:** Jessica A Altamirano, Prerak Shukla, Steven K Barnett

**Affiliations:** CAN Community Health, Miami, Florida; CAN Community Health, Miami, Florida; CAN Community Health, Miami, Florida

## Abstract

**Background:**

Lenacapravir (LEN) is the first-in-class long-acting capsid inhibitor approved by the FDA in December 2022 for the treatment of multi-drug resistant HIV in adults. It is administered by subcutaneous injection every six months in combination with other antiretrovirals (ARV). It is indicated for heavily treatment-experienced people with HIV (PWH). We assessed the use of long-acting LEN within the CAN Community Health Network.

**Figure:**
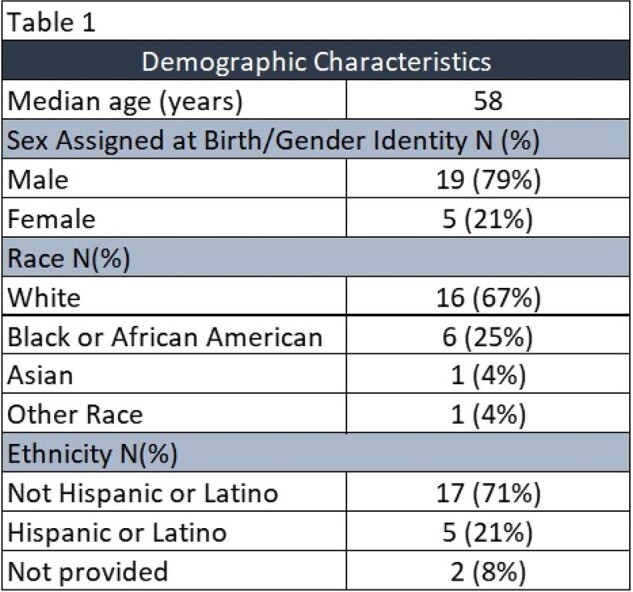

**Methods:**

A retrospective analysis of electronic medical records within the CAN Community Health Network of 23 clinics located throughout six US states. People with HIV who received one or more injections of LEN for treatment between December 2022 and April 2024 were included. Demographic characteristics, HIV viral load, CD4 count and composition of pre-LEN ARV regimens were studied.

**Figure:**
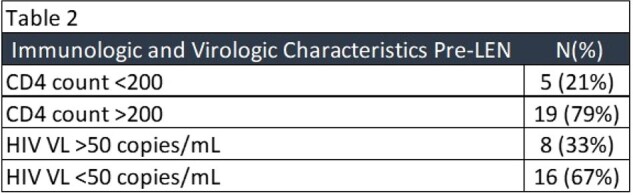

**Results:**

Twenty-four PWH were identified as receiving one or more injections of LEN between December 2022 and April 2024. The median age was 58 years; 79% were male, 67% White and 71% Non-Hispanic (Table 1). Mean number of injections received was 2. Pre-LEN HIV VL was >50 copies/mL in 8 PWH (33%) and pre-LEN HIV VL was < 50 copies/mL in 16 PWH (67%). Five PWH (21%) had a pre-LEN CD4 count < 200 and 19 PWH (79%) had a pre-LEN CD4 count >200. Post-LEN HIV VL decreased in all eight patients with HIV VL >50 copies/mL (Table 2).

Pre-LEN ARV regimens included a median of 4 active agents with 92% of them containing integrase inhibitors (INSTIs), 71% NRTIs and 58% protease inhibitors (PIs) (Table 3). Reasons for switching to a LEN-combination regimen in those with HIV VL < 50 included: simplification, side effects or intolerance to oral ARVs and non-adherence. Three (13%) patients stopped LEN due to injection site reactions (ISRs), 1 patient (4%) stopped due to side effects, 2 patients (8%) were lost to care and 1 patient (4%) was switched to an oral alternative due to travel.

**Figure:**
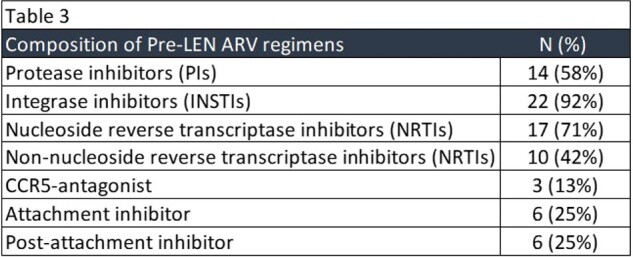

**Conclusion:**

The majority of PWH who were switched to LEN-combination regimens were older males with HIV VL < 50 copies/mL. Reasons for switching included simplification, side effects to other ARVs and non-adherence. Of those with HIV VL > 50 copies/mL prior to switching to a LEN-combination regimen, all had a decrease in their HIV VL. The most common reason for discontinuation was ISRs. Sample size was small and a longer duration of follow-up is needed.

**Disclosures:**

**Jessica A. Altamirano, MD, FIDSA**, Gilead Sciences: Speaker's Bureau

